# 2-fluoro-1-methylpyridinium p-toluene sulfonate: a new LC-MS/MS derivatization reagent for vitamin D metabolites

**DOI:** 10.1016/j.jlr.2023.100409

**Published:** 2023-07-03

**Authors:** Anastasia Alexandridou, Dietrich A. Volmer

**Affiliations:** Bioanalytical Chemistry, Humboldt University Berlin, Berlin, Germany

**Keywords:** vitamin D_3_ metabolites, 25-hydroxyvitamin D_3_, chemical derivatization, FMP-TS, LC-MS/MS

## Abstract

Vitamin D analysis by MS faces several analytical challenges, including inefficient ionization, nonspecific fragmentation, interferences from epimers, isomers, and isobars, as well as very low concentration levels. In this study, we used 2-fluoro-1-methylpyridinium (FMP) p-toluene sulfonate for derivatization of vitamin D3 metabolites to increase detection sensitivity and allow for full chromatographic separation of vitamin D isomers and epimers. UHPLC-MS/MS was used for measurement of five vitamin D3 metabolites in human serum. Compared with Amplifex and 4-phenyl-1,2,4-triazolin-3,5-dion, the FMP p-toluene sulfonate reaction required less time to be performed. The method was optimized and validated to ensure accuracy, precision, and reliability. In-house and commercial quality control samples were used to assure the quality of the results for 25-hydroxyvitamin D3. The method showed very good linearity and intraday and interday accuracy and precision; coefficients of determination (r^2^) ranged between 0.9977 and 0.9992, relative recovery from 95 to 111%, and coefficient of variation from 0.9 to 11.3. Stability tests showed that the extracted derivatized serum samples were stable for 24 h after storage at −20°C; 24,25-dihydroxyvitamin D3 and 1,25-dihydroxyvitamin D3-FMP derivatives were stable for 1 week at −80°C. The method was applied to samples of healthy individuals for quantitative determination of vitamin D3, the two epimers of 25-hydroxyvitamin D3 and 24,25-dihydroxyvitamin D3.

## Graphical abstract

**Figure:**
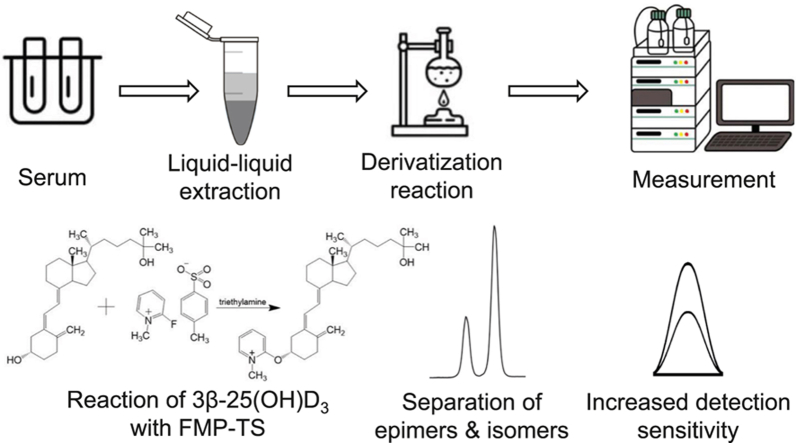

Determining the vitamin D status of individuals requires accurate and precise quantification of 25-hydroxyvitamin D (25(OH)D) concentration levels (1). Currently, LC-MS/MS is considered the “gold standard” technique for this purpose (2, 3). As other vitamin D metabolites have also been proposed as markers for different pathological conditions (4), it is important to develop methods for simultaneous measurement of multiple vitamin D_3_ metabolites with adequate precision, accuracy, and sensitivity.

Analytical method development for vitamin D metabolites using MS is challenging, however, because of the poor ionization efficiencies of these molecules and the low concentration levels in biological samples (5). To overcome these difficulties, optimized sample preparation methods, including chemical derivatization, can improve analyte recovery from the matrix, eliminate coexisting interferences, and increase ionization efficiency (6). In addition, derivatization shifts masses to higher *m/z* values, which generally exhibit less isobaric noise and often generate more specific fragmentation patterns during MS/MS analysis. Moreover, derivatization can improve chromatographic separation of analytes, which is particularly important for vitamin D metabolites, as separation of isomers and epimers can be challenging. Derivatization of vitamin D compounds often yields multiple products for each metabolite, however, which will then generate several peaks during chromatography (7).

For vitamin D, derivatization using Cookson-type reagents is well established, including 4-phenyl-1,2,4-triazoline-3,5-dione (8, 9, 10, 11, 12), Amplifex (13, 14, 15), 2-nitrosopyridine, 4-(4′-dimethylaminophenyl)-1,2,4-triazoline-3,5-dione (16), and 4-[2-(6,7-dimethoxy-4-methyl-3-oxo-3,4-dihydroquinoxalyl) ethyl]-1,2,4-triazoline-3,5-dione (17). These reagents are very specific as they target the unique s-cis moiety of vitamin D compounds.

In contrast, reagents such as isonicotinoyl chloride and Girard P (18, 19) target the much less specific hydroxyl groups. Vitamin D metabolites possess one or more -OH groups and, depending on stereochemical hindrances and reagent concentration, usually lead to between one and three products for each molecule. Diels-Alder and hydroxyl group derivatizations can also be combined, as described by Higashi *et al.* (20). Depending on the polarization of the C–OH bond, different synthetic strategies are required; for example, by nucleophilic substitution or via initial alkene formation and subsequent derivatization. Dehydration is required for the latter, requiring the -OH group to be located next to a quaternary carbon (21).

In this study, 2-fluoro-1-methylpyridinium p-toluene sulfonate (FMP-TS) was used for hydroxyl group derivatization. FMP-TS is a 2-halopyridinium salt used for synthesis of esters, amides, and thiol esters (22, 23). As hydroxyl groups are poor leaving groups, reactions via S_N_2 are not very effective (24), but reactions employing oxygen as nucleophile can be used to produce ionizable groups or add preionized groups via nucleophilic substitution. It was shown that primary and secondary hydroxyl groups can be converted to *N*-methylpyridyl ether salts by nucleophilic displacement of FMP-TS by oxygen (21). Bald (25) demonstrated that the reaction takes place in various solvents in the presence of triethylamine (TEA).

In this study, we present a new derivatization method for LC-MS/MS quantification of five major vitamin D_3_ metabolites in serum samples using FMP-TS after liquid-liquid extraction (LLE). The derivatization reaction was very quick, easy to perform, and inexpensive. We describe optimization of various parameters of the derivatization reaction as well as validation of the entire assay. To the best of our knowledge, this is the first time that FMP-TS has been used for measurement of vitamin D_3_, 3β-25-dihydroxyvitamin D_3_ (3β-25(OH)D_3_), 3α-25-dihydroxyvitamin D_3_ (3α-25(OH)D_3_), 1,25-dihydroxyvitamin D_3_ (1,25(OH)_2_D_3_), and 24,25-dihydroxyvitamin D_3_ (24,25(OH)_2_D_3_) in serum samples. It has been previously reported as derivatization reagent for estrogen in water, human plasma, and serum (26, 27, 28).

## Materials and methods

### Chemicals, reagents, and materials

FMP-TS (technical grade ≥90%), 2-hydroxy-1-methylpyridinium p-toluene sulfonate, technical grade ≥90%) and 3α-25(OH)D_3_ were purchased from Sigma-Aldrich (Steinheim, Germany), 3β-25(OH)D_3_ and 1,25(OH)_2_D_3_ from Cayman Chemical (Ann Arbor, MI), and (24R)-24,25(OH)_2_D_3_ and vitamin D_3_ from Toronto Research Chemicals (Toronto, ON, Canada). The isotopically labeled internal standards (ISs) 3α-25(OH)D_3_-[26,26,26,27,27,27-d6] and 24,25(OH)_2_D_3_-[26,26,26,27,27,27-d6] were from Endotherm (Saarbrücken, Germany), 1,25(OH)_2_D_3_-[26,26,26,27,27,27-d6] from Chemaphor (Ottawa, ON, Canada), 3β-25(ΟΗ)D_3_-[26,26,26,27,27,27-d6] monohydrate from IsoSciences (Ambler, PA), and vitamin D_3_-[6,19,19-d3] from Sigma-Aldrich.

Acetonitrile (ACN), methanol, and ethyl acetate were of UHPLC-MS grade and obtained from CHEMSOLUTE (Th. Geyer, Renningen, Germany); formic acid (97%) was from Alfa Aesar (Karlsruhe, Germany). TEA (≥99.5%, for synthesis) was from Carl Roth (Karlsruhe, Germany), and trimethylamine (TMA) (13% in ACN, 2 mol/l) was from Tokyo Chemical Industry (Tokyo, Japan). A Millipore (Bedford, MA) Direct-Q8 purification system was used to generate organic-free water.

Human vitamin D_3_-free serum and the molecular sieve beads (0.3 nm, sodium aluminum silicate, ∼2 mm/∼10 mesh) were from Sigma-Aldrich (VD-DDC Mass Spect Gold® serum). Commercially available quality control (QC) serum samples, ClinChek® Serum Control for 25-OH vitamins D_2_/D_3_, lyophilized, levels I and II, were obtained from RECIPE (Munich, Germany).

### Preparation of standard solutions and QC samples

Stock standard solutions of vitamin D_3_ metabolites were prepared in methanol at 1 mg/ml and their respective isotope IS at 1 μg/ml. All solutions were stored at −20^°^C.

Human vitamin D_3_ free serum was spiked with vitamin D_3_ standard solutions to produce QCs and samples for calibration curves for each analyte (Table 1). Concentration of QCs was prepared at three levels representing samples with high, medium, and low concentration levels of the five investigated vitamin D_3_ metabolites.

**Table 1:** Concentrations of metabolites (ng/ml) in QC samples and samples for calibration curves

QC/Cal	24,25(OH)_2_D_3_	1,25(OH)_2_D_3_	3α-25(OH)D_3_	3β-25(OH)D_3_	D_3_^a^
Concentration, ng/ml					
QC low	0.5	0.5	0.5	8	8.7
QC medium	8	5	8	50	50.7
QC high	35	9	18	90	90.7
Cal 0	0	0	0	0	0.7
Cal 1	0.5	0.4	0.1	2	2.7
Cal 2	1	0.6	0.8	4	4.7
Cal 3	5	1	2	10	10.7
Cal 4	10	2	4	20	20.7
Cal 5	20	4	6	40	40.7
Cal 6	30	6	10	60	60.7
Cal 7	40	8	15	80	80.7
Cal 8	50	10	20	100	100.7

a VD-DDC Mass Spect Gold® vitamin D-free serum contained blank concentration of vitamin D_3_ at 0.7 ng/ml.

### Biological samples

The method was applied to 12 human plasma samples from healthy individuals and approved by the Local Research Ethics Committee (Charité Universitätsmedizin Berlin, ethics number EA2/176/19). Written informed consent was provided by all participants. The human study reported in this article abided by the Declaration of Helsinki principle.

### Derivatization

Vitamin D_3_-free serum samples were spiked with 3α-25(OH)D_3_ at 12 ng/ml, 3β-25(OH)D_3_ at 50 ng/ml, 1,25(OH)_2_D_3_ at 5 ng/ml, (24R)-24,25(OH)_2_D_3_ at 25 ng/ml, vitamin D_3_ at 50 ng/ml, 3α-25(OH)D_3_-d6 at 6 ng/ml, 3β-25(OH)D_3_-d6 at 20 ng/ml, 1,25(OH)_2_D_3_-d6 at 6 ng/ml, 24,25(OH)_2_D_3_-d6 at 6 ng/ml, and vitamin D_3_-d3 at 10 ng/ml. Subsequently, 100 μl of serum sample were pretreated using the sample preparation protocol described below (Extraction section), followed each time by derivatization. Two independent samples were prepared per investigated optimization parameter, and samples were measured in duplicate.

The reagent was dissolved in regular ACN and dry ACN (5 mg/ml) to investigate if the presence of water affected the reaction. Dry ACN was prepared by using a bead-type molecular sieve (0.3 nm, sodium aluminum silicate). Furthermore, two bases (TEA and TMA) were examined in their suitability to promote the derivatization reaction. Base concentration was optimized by addition of 1, 2, and 4% v/v. The concentration of the reagent was studied at 1, 5, 10, and 20 mg/ml. After optimization of the added components, incubation parameters were examined; viz., incubation temperatures at 22^°^C (room temperature), 30, 40, and 50^°^C; incubation time was compared at 5, 10, 15, 20, and 30 min.

Based on these optimization experiments, the final protocol was as follows: FMP-TS solution was freshly prepared in dry ACN at 5 mg/ml containing 1% TEA; 50 μl were added to the dried sample residue. The mixture was vortexed for 15 s and incubated for 15 min at 30^°^C. Subsequently, the reaction was quenched using 50 μl of methanol. After solvent evaporation to dryness, the sample was reconstituted using 100 μl of a mixture of 44/56 ACN/H_2_O v/v (initial composition of mobile phase) prior to LC injection.

### Extraction

Prior to sample preparation, 10 μl of a mixture of the five ISs were added to 100 μl of sample to give the following concentrations: 3α-25(OH)D_3_-d6 at 6 ng/ml, 3β-25(OH)D_3_-d_6_ at 20 ng/ml, 1,25(OH)_2_D_3_-d_6_ at 6 ng/ml, 24,25(OH)_2_D_3_-d6 at 6 ng/ml, and vitamin D_3_-d_3_ at 10 ng/ml. Protein precipitation, two-step LLE, and derivatization were used, based on a previous method reported by us (8) and a modified version described by Alexandridou *et al.* (29) for 100 μl of serum sample. Briefly, protein precipitation was achieved by adding 250 μl of ACN to 100 μl of sample, followed by 1 min of vortexing and 15 min of centrifugation at 10,000 rpm. A Concentrator plus/Vacufuge® plus (Eppendorf, Hamburg, Germany) was used to evaporate the supernatant to dryness after its transfer to a new vial. For LLE, 100 μl of water and 200 μl of ethyl acetate were added to the dry residue followed by 30 s of vortexing and 5 min of centrifugation at 10,000 rpm. The organic phase was transferred to a new vial, and the aqueous phase was re-extracted by adding 200 μl of ethyl acetate. The two organic fractions were combined and evaporated to dryness prior derivatization.

### LC-MS/MS

Chromatographic separations were performed on a 1290 Infinity II LC system (Agilent, Santa Clara, CA) after injection of 5 μl of sample. The autosampler was set to 10^°^C. A Phenomenex (Torrance, CA) Kinetex® 2.6 μm F5 (pentafluorophenyl) 100 Å column (100 × 2.1 mm) was used in combination with a mobile phase consisting of (A) water (+0.1% formic acid) and (B) ACN (+0.1% formic acid). The column temperature was set to 35^°^C, and the flow rate was 0.4 ml/min. An isocratic step of 44% B was held for the first 4 min, followed by the following gradient steps: increase to 49% B in 2.5 min, steep increase to 70% in 0.1 min, and increase from 70% to 82% B in 3.5 min. Finally, B was held at 100% for 2 min, before 2 min re-equilibration at the initial conditions. The total time of analysis was 14 min.

The UHPLC system was coupled to a Sciex (Concord, ON, Canada) QTRAP 6500+ quadrupole-linear ion trap MS. Electrospray ionization was conducted in positive ion mode using a Turbo-V source using multiple reaction monitoring. Ion source and multiple reaction monitoring parameters were optimized to achieve the highest detection sensitivity (supplemental Table S1).

Dwell times were chosen to obtain 12 to 20 data points across chromatographic peaks. Analyst software (Sciex), version 1.7, was used for data acquisition, whereas data analysis was performed using MultiQuant (Sciex), version 3.0.3.

## Method validation

### Recovery

Extraction recoveries were determined for the five metabolites at three concentration levels; low, medium, and high; results are presented in the Results and Discussion section.

### Linearity

Samples for calibration curves were prepared using vitamin D-free serum, spanning the expected concentration range in the blood samples. Eight calibrants were prepared for each metabolite; each concentration was measured in triplicate. Moreover, a blank sample without the investigated analyte or the respective IS, and a “zero” sample containing no analyte, but IS was prepared for every calibration curve. The coefficient of determination (*r*^2^) was used to evaluate the linearity for every analyte.

### Accuracy and precision

Accuracy and precision were determined for all investigated metabolites using spiked serum samples (QCs) at three different concentration levels (low, medium, and high) in the presence of IS. QCs were prepared independently from the samples used for calibration curves. Three independent samples were prepared per concentration level and were measured in triplicate to evaluate intraday or within-run accuracy and precision. Interday or between-run accuracy and precision were validated by three independent runs within 1 week of the QCs above (three samples analyzed in triplicate). Relative recovery (*R*%) (Equation 1) was used to express accuracy, whereas precision was expressed as coefficient of variation (%CV) (Equation 2).

(Eq. 1) $$R\%=\frac{Mean\;of\;experimental\;concentrations}{Nominal\;concentration}\times 100$$

(Eq. 2) $$\%CV=\frac{Mean\;standard\;deviation\;of\;experimental\;concentrations}{Mean\;value\;of\;experimental\;concentrations}\times 100$$

ClinChek® Serum Control-certified reference standards were used to evaluate the accuracy and precision for 25(OH)D_3_ at two different concentration levels (low and high). Three samples of the different concentration levels were prepared and analyzed three times each.

Accuracy was considered acceptable when the mean concentration of the QCs was within 15% of the nominal concentration. An acceptable precision required CV% values not exceeding 15% for the QC samples.

### Limit of detection and lower limit of quantitation

To determine limit of detection (LOD) and lower limit of quantitation (LLOQ), vitamin D-free serum was spiked at decreasing concentration levels; these samples were measured in triplicate. The lowest concentration of analyte with signal-to-noise (*S/N*) ratio >3 determined LOD. LLOQ was defined as the lowest calibrant concentration, which could be quantified with acceptable accuracy and precision according to the “Guideline on Bioanalytical Method Validation” by the European Medicines Agency (https://www.ema.europa.eu/en/documents/scientific-guideline/guideline-bioanalytical-method-validation_en.pdf) and the guidelines of the Food and Drug Administration for bioanalytical method validation (https://www.fda.gov/files/drugs/published/Bioanalytical-Method-Validation-Guidance-for-Industry.pdf).

### Stability of the derivatization products

The stability of the derivatized analytes was examined in order to investigate if processed samples could still be measured after remaining in the autosampler for 24 h at 10^°^C and after storage for 24 h at −20^°^C. Long-term stability of the derivatization products was also investigated, including storage for 1 week at −80^°^C. In every experiment, three concentration levels were measured (low, medium, and high), and three independent samples were prepared and measured in triplicate. Freshly prepared samples of each concentration level were immediately measured to establish time 0 responses (*t* = 0). Stability was considered acceptable if the mean concentration of each level was between ±15% of the nominal concentration.

## Results

### Chromatography and MS of FMP-TS products

**Fig. 1:**
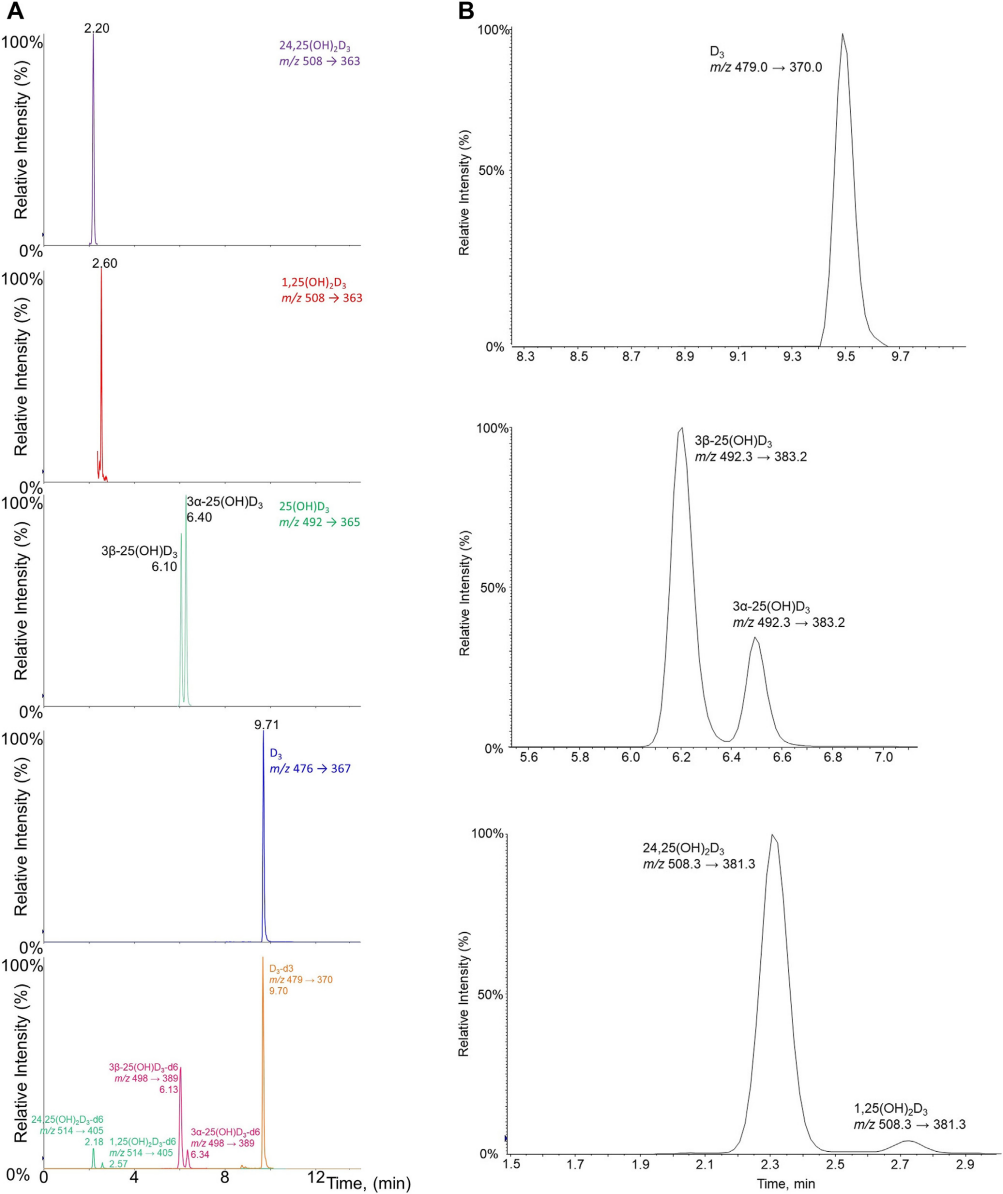
A: Chromatographic separation of the investigated FMP-derivatized vitamin D_3_ metabolites and their respective IS using a pentafluorophenyl stationary phase and (A) H_2_O + 0.1% formic acid, (B) ACN + 0.1% formic acid as mobile phase. B: Chromatographic separation of the investigated FMP-vitamin D_3_ metabolites in human serum.

All FMP-TS products for the investigated vitamin D metabolites were readily resolved by LC using the instrumental conditions described in the Materials and methods section. Figure 1A shows extracted ion chromatograms of derivatized standards and ISs. For the epimers, baseline separation was achieved for derivatized 25(OH)D_3_ with calculated resolution *R*_*s*_ = 2.0 (Fig. 1A, green traces). Figure 1B illustrates extracted ion chromatograms, showing that all derivatized analytes can be readily separated from interferences in human serum. Total chromatographic run time was 14 min; total injection-to-injection cycle time was 14.2 min.

The common vitamin D metabolites offer up to three -OH groups for reaction with FMP-TS via nucleophilic substitution; viz., the native -OH group of vitamin D at C-3 plus two metabolic oxidations. However, only single addition of FMP was observed for 1,25(OH)_2_D_3_, with accompanying ions for [M+92]^+^ at *m/z* 508 and [M+92-H_2_O]^+^ at *m/z* 490 (after loss of water). The same ions were seen for 24,25(OH)_2_D_3_, with an additional [M+184]^2+^ ion at *m/z* 300, after reaction of the second -OH group. For both 1,25(OH)_2_D_3_ and 24,25(OH)_2_D_3_, the ion with the highest abundance was [M+92]^+^ at *m/z* 508. The fragmentation patterns for all derivatized metabolites were very similar in comparison to the nonderivatized molecules; that is, after C_6_H_7_ON loss, one H_2_O molecule was cleaved off for monohydroxylated species and two H_2_O molecules for the dihydroxylated species. Similarly, 3α-25(OH)D_3_ and 3β-25(OH)D_3_ reacted only at one -OH group, with resulting precursor [M+92]^+^ ion at *m/z* 492; for vitamin D_3_, [M+92]^+^ was at *m/z* 476 (supplemental Table S1).

The sensitivity gain of the method was investigated from spiked serum samples after measuring derivatized and nonderivatized samples. Serum samples were preferred over standards in solvent because matrix effects were included in this comparison. The results are summarized in Table 2. Gains were expressed as relative peak areas, calculated as peak area ratio of derivatized to nonderivatized analyte.

**Table 2:** Relative peak area ratios for the five metabolites

Metabolite	Relative peak area (A_der_/A_non-der_)
24,25(OH)_2_D_3_	12
1,25(OH)_2_D_3_	3
3β-25(OH)D_3_	33
3α-25(OH)D_3_	30
D_3_	285

### Derivatization optimization

The reaction conditions were optimized using spiked serum samples containing the different metabolites at concentration levels comparable to human samples (see Materials and methods section). Also, blank serum samples were used to ensure the absence of coeluting isomers or isobars for analytes and ISs. Several experimental conditions were investigated consecutively to enhance the detection sensitivity of the method: *1*) type of solvent and the presence of moisture in the reaction solvent; *2*) added base and concentration, *3*) FMP-TS concentration, and *4*) incubation temperature and incubation time.

1. Bald (25) investigated the reaction of FMP-TS with thiols in various organic solvents and observed no effects on the reaction. However, Lin *et al.* (28) found that FMP-TS was not adequately soluble in dichloromethane and therefore changed to ACN. ACN was shown to be the preferred solvent for FMP-TS reactions, and we further investigated whether the presence of water in the solvent affected the reaction. Detection sensitivity improved ≈1.3× for dry ACN for all metabolites over regular ACN except for 1,25(OH)_2_D_3_-FMP, which was unaffected.
2. TEA and TMA were added to dry ACN as bases at 1% (v/v) to examine their impact on the reaction. Adding TEA changed the FMP-TS reaction solution to a yellow color (supplemental Fig. S1) and increased the gain for all analytes *ca.* 1.3-fold over TMA, except for 1,25(OH)_2_D_3_-FMP, where TMA exhibited ≈1.2× response gain over TEA. TEA concentration was then increased from 1, 2–4% v/v in dry ACN. Higher base concentration levels (2 and 4%) only promoted 1,25(OH)_2_D_3_-FMP formation (up to 1.6× for 2% and 1.8× for 4% compared with 1%), whereas it negatively affected the other metabolites. Specifically, 1% base concentration improved detection sensitivity for 24,25(OH)_2_D_3_-FMP and 3β-25(OH)_2_D_3_-FMP 2-fold, 35-fold for 3α-25(OH)_2_D_3_-FMP, and 3-fold for D_3_-FMP, as compared with 4%. As a result, 1% was chosen for TEA in all subsequent experiments.
3. The experiments above were conducted at FMP-TS concentration of 5 mg/ml. We also systematically investigated the impact of FMP-TS concentration on the reaction outcome. The results are shown in Figure. 2. Increasing reagent concentration from 1 to 5 mg/ml increased peak areas for all metabolites, whereas further increases provided only detrimental effects for 1,25(OH)_2_D_3_, 24,25(OH)_2_D_3,_ and vitamin D_3_. The 25(OH)D_3_ epimer slightly increased further upon increased FMP-TS concentration up to 20 mg/ml.

**Fig. 2:**
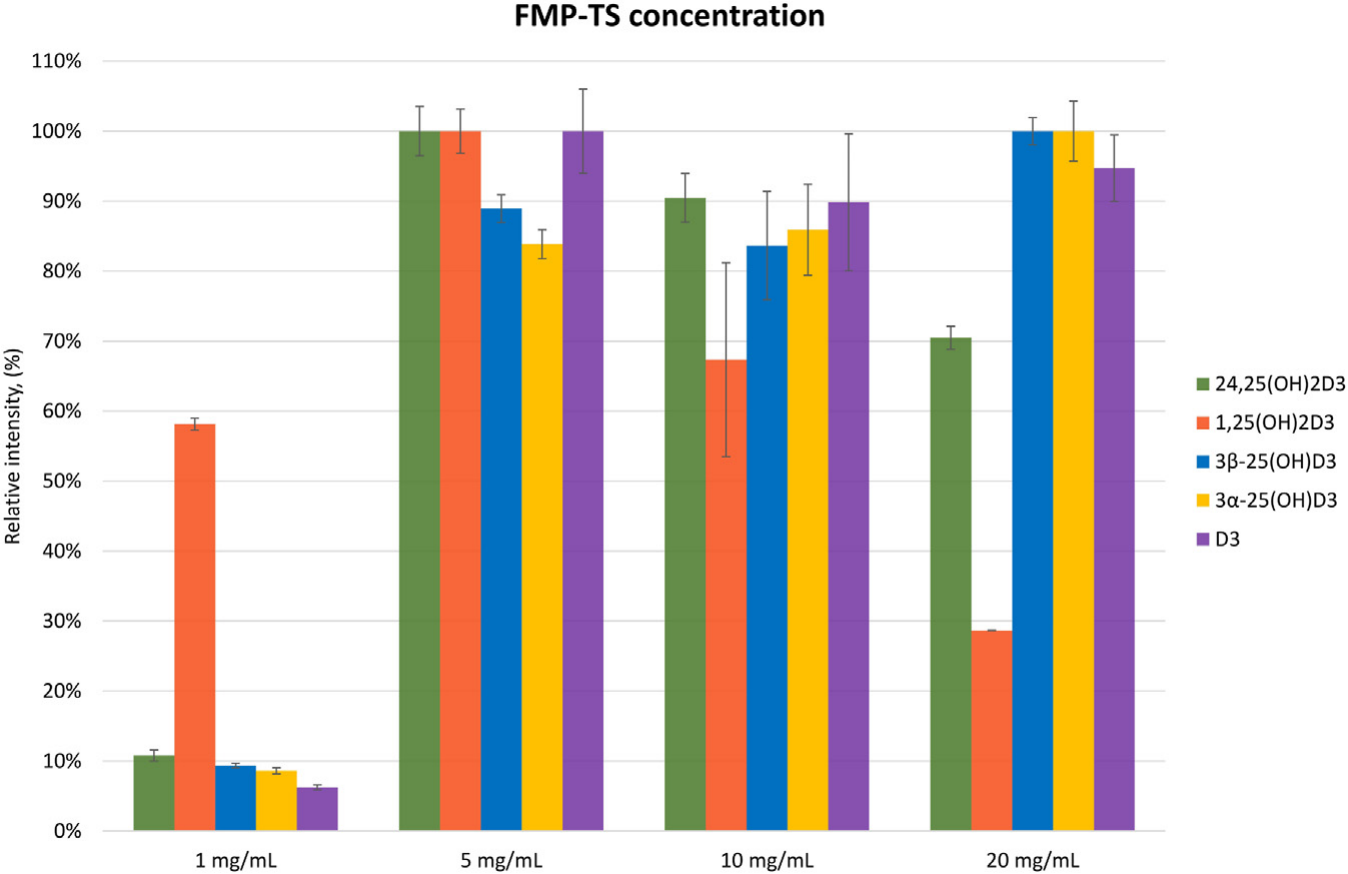
Optimization of FMP-TS concentrations in dry ACN (+TEA, 1% v/v) at 40^°^C for 15 min (standard deviation is shown in the bar chart).
4. In the above experiments, incubation time and temperature were 15 min and 40^°^C, respectively. The reaction yields improved when increasing the temperature from 22^°^C (room temperature) to 30^°^C. Only 1,25(OH)_2_D_3_ exhibited further increases at temperatures up to 50^°^C, whereas other metabolites showed decreased yields for temperatures above 30^°^C.

**Fig. 3:**
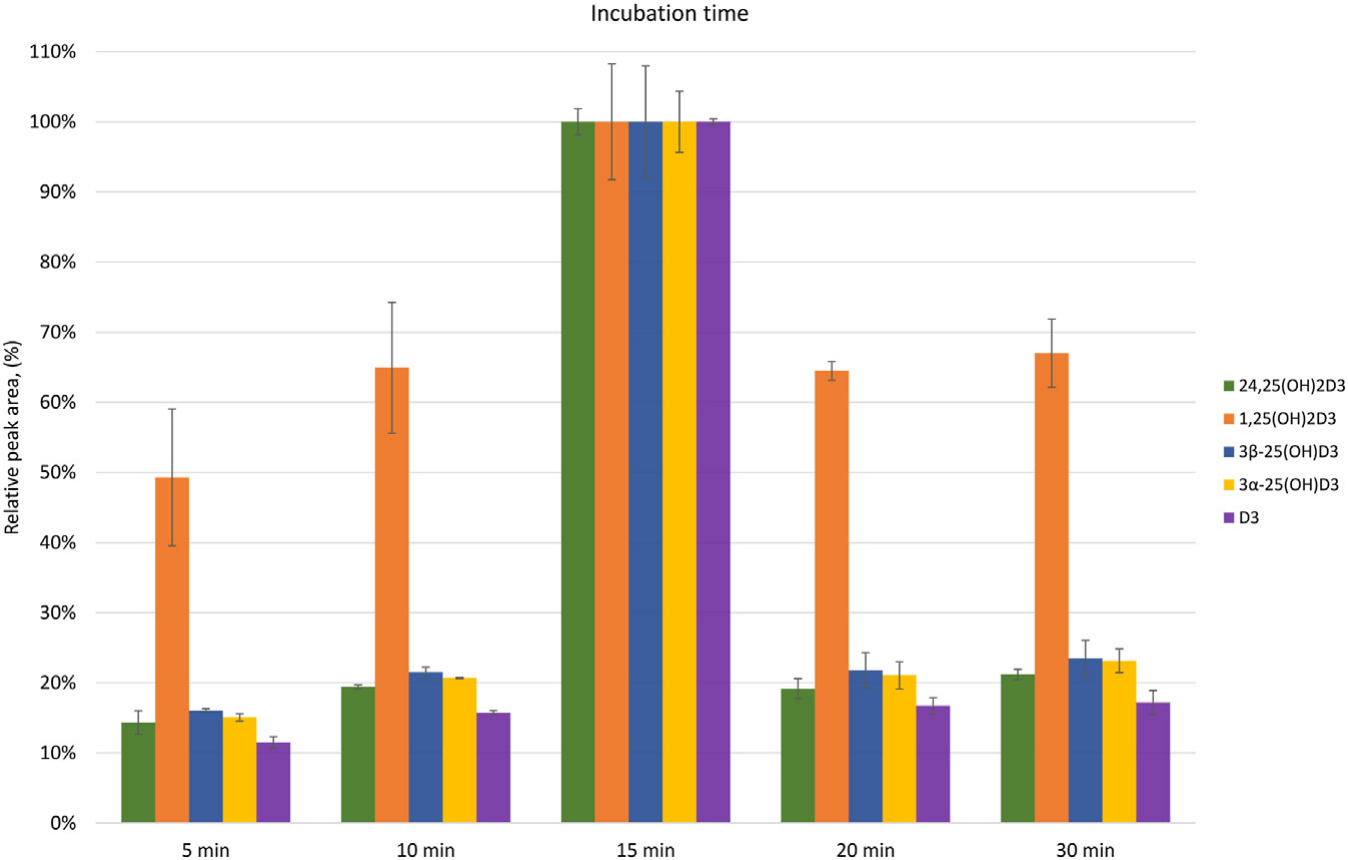
Optimization of incubation time (following optimization of all other experimental conditions; the highest individual yields were each normalized to 100%; standard deviation is shown in the bar chart).

Finally, Fig. 3 demonstrates the effects of incubation time on reaction yields in our experiments. Increasing incubation time from 5 to 15 min strongly improved yields for all metabolites, which then declined again for longer reaction times (up to 30 min), most likely as a result of degradation reactions. We chose the optimum yield at 15 min for our assay. Any variability of yields as a result of small variations of the incubation time is compensated by the equally affected IS, thus maintaining the accuracy. Equally, as LLOQ values in this study were determined based on the precision of the assay and thus gave much higher values than LLOQs based on signal-to-noise ratios (e.g., 10× S/N), absolute ion currents for all metabolites were always high, even at LLOQ, regardless of the incubation time.

### Method validation

The method was validated by means of guidelines of the Food and Drug Administration for bioanalytical method validation (https://www.fda.gov/files/drugs/published/Bioanalytical-Method-Validation-Guidance-for-Industry.pdf) and the European Medicines Agency (https://www.ema.europa.eu/en/documents/scientific-guideline/guideline-bioanalytical-method-validation_en.pdf). Here, recovery, linearity, accuracy, precision, LOD, LLOQ, as well as stability were determined according to these guidelines.

### Recovery

The extraction recovery was investigated for every metabolite at three different concentration levels (low, medium, and high) to ensure that extraction is efficient and reproducible. Percentage extraction recovery (*R*%) was calculated as follows:

$$R\%=\frac{E_{BE}{/E}_{IS}}{E_{AE}{/E}_{IS}}\times 100$$

E_BE_: peak area of investigated metabolite spiked in the sample prior to extraction.

E_AE_: peak area of investigated metabolite spiked after extraction.

E_IS_: peak area of the IS of each metabolite.

Vitamin D-free serum samples were spiked with a mixture of the analytes in methanol preextraction and postextraction in triplicate, followed by derivatization and analysis. Mean values of the three independent samples were used (Table 3). Satisfactory *R*% values between 81% and 99% were achieved, demonstrating that the five metabolites were adequately extracted across the investigated concentration levels.

**Table 3:** Extraction recovery (*R*%) of the investigated metabolites at three concentration levels in serum

Metabolite	Concentration (ng/ml)	*R%*	*r*^2^	LOD (ng/ml)	LLOQ (ng/ml)
24,25(OH)_2_D_3_	6	92	0.9992	0.1	0.5
	25	85			
	45	97			
1,25(OH)_2_D_3_	0.8	95	0.9992	0.2	0.4
	5	81			
	9	99			
3β-25(OH)D_3_	8	93	0.9992	0.05	2
	50	90			
	90	93			
3α-25(OH)D_3_	5	94	0.9990	0.02	0.1
	12	88			
	18	93			
D_3_	8	89	0.9977	—	0.7
	50	82			
	90	85			

Linearity was expressed as coefficient of determination (r2), LOD, and LLOQ.

### Linearity

Simple linear regression was chosen as calibration model only for 24,25(OH)_2_D_3_ using the least squares method. For the other metabolites, weighted factor 1/x using least squares was applied. The coefficient of determination (*r*^2^) was used to evaluate linearity for each analyte. Table 3 shows *r*^2^ values, calibrant concentrations, and working range for the investigated metabolites.

### Accuracy, precision, and QC samples

The developed method was accurate and precise, complying with the criteria outlined in the used guidelines. Intraassay accuracy, expressed as recovery %, ranged from 95% (vitamin D_3_) to 111% (24,25(OH)_2_D_3_) and interassay accuracy ranged from 96% (3α-25(OH)D_3_) to 111% (24,25(OH)_2_D_3_). Precision was acceptable within a day, since within the same analytical batch, %CV values ranged from 0.9% (1,25(OH)_2_D_3_) to 11.3% (vitamin D_3_). Within the period of 1 week, interday precision ranged from 2.4% (1,25(OH)_2_D_3_ and 24,25(OH)_2_D_3_) to 9.9% (3β-25(OH)D_3_). Detailed information on accuracy and precision of this new method is summarized in Table 4.

**Table 4:** Concentration of QC samples, intraday and interday accuracy, and precision

Accuracy & precision		Intraday		Interday	
Analyte	Concentration (ng/ml)	*R*%	%CV	*R*%	%CV
24,25(OH)_2_D_3_	0.5	111	2.1	111	2.4
	8	101	1.9	99	2.5
	35	101	3.1	98	5.8
1,25(OH)_2_D_3_	0.5	102	2.7	102	2.4
	5	105	0.9	107	2.4
	9	100	1.8	103	2.9
3β-25(OH)D_3_	8	97	8.1	99	9.9
	50	104	1.4	102	5.0
	90	96	6.2	100	4.9
3β-25(OH)D_3_ QC	RECIPE level I: 14.9	97	1.7	—	—
	RECIPE level II: 42.0	100	2.7	—	—
3α-25(OH)D_3_	0.5	102	4.0	96	6.2
	8	99	2.3	96	3.3
	18	100	3.3	101	4.6
D_3_	8.7	95	7.3	98	9.2
	50.7	103	8.6	100	9.2
	90.7	102	11.3	102	6.0

Commercial ClinChek® Serum Control was used as additional measure to evaluate accuracy and precision for 3β-25(OH)D_3_ at two different concentration levels. These samples were analyzed during the intraday evaluation phase for accuracy and precision as well as during the analyses of human samples (Table 4). The resulting *R*% (97–100%) and %CV (<3%) values demonstrate that the method’s performance was excellent for the vitamin D status marker.

### LOD and LLOQ

For the native vitamin D_3_ species, LLOQ was defined by the blank concentration contained in the commercial vitamin D-free serum used in this study. By standard addition, we found that VD-DDC Mass Spect Gold® serum contained vitamin D_3_ at 0.7 ng/ml, which is within specifications for this product (vitamin D_3_, ≤1 ng/ml). For this reason, no LOD was determined for vitamin D_3_. To determine LLOQ of the other metabolites, three samples of the lowest calibrator were analyzed and measured in triplicate to demonstrate that precision (CV% <20%) and accuracy (80–120%) criteria were met. Table 3 summarizes LOD and LLOQ values determined for the investigated compounds.

### Stability of derivatization products

Short-term stability (24 h at −20^°^C) and long-term stability (1 week at −80^°^C) were investigated for the derivatized metabolites in the processed matrix. The obtained concentrations were compared with the nominal concentrations using a freshly prepared calibration curve for each analyte. The results are summarized in Table 5. The stabilities of the extracts in the autosampler were also investigated (supplemental Table S2) and demonstrate that the processed samples can safely remain in the autosampler for 24 h at 10^°^C until measurement.

**Table 5:** Short-term and long-term stability of FMP products of vitamin D_3_ metabolites in serum extract

Stability		*t* = 0		24 h, −20^°^C		1 week, −80^°^C	
Analyte	Spiked concentration (ng/ml)	Mean accuracy (R%)	%CV	Mean accuracy (R%)	%CV	Mean accuracy (R%)	%CV
24,25(OH)_2_D_3_	0.5	99	9.0	107	4.6	97	8.0
	8	95	1.3	91	2.0	87	1.5
	35	94	5.2	90	2.1	85	8.1
1,25(OH)_2_D_3_	0.5	107	4.4	104	2.9	95	4.4
	5	99	3.8	93	1.5	93	3.5
	9	95	1.4	93	1.8	92	1.2
3β-25(OH)D_3_	8	110	3.6	98	3.0	77	9.3
	50	106	2.6	93	7.4	82	1.9
	90	102	1.4	90	3.2	75	4.7
3α-25(OH)D_3_	0.5	102	4.0	97	2.4	76	7.1
	8	99	2.3	90	4.5	75	3.0
	18	100	3.3	95	2.8	79	4.6
D_3_	8.7	105	2.2	99	5.8	77	9.8
	50.7	108	5.2	101	5.6	85	7.7
	90.7	103	3.9	99	4.0	74	8.2

All five derivatized vitamin D_3_ metabolites were accurately measured at all concentration levels (R% ranged between 90 and 107%) with good precision (%CV ≤7.4) after storage for 24 h at −20^°^C. However, only 24,25(OH)_2_D_3_ and 1,25(OH)_2_D_3_ products meet acceptable criteria after storage for 1 week at −80^°^C (R% ranged between 85 and 97%). For the other metabolites, the mean concentration ranged between −15% and −26% of the nominal concentration. Further information on longer storage periods (1 and 3 month storage at −20^°^C) was reported previously and is in agreement with the results presented here (29).

### Application to serum samples from healthy individuals

The newly developed method was applied to 12 plasma samples of healthy individuals. Vitamin D_3_, 24,25(OH)_2_D_3_, 3α-25(OH)D_3_, and 3β-25(OH)D_3_ were successfully quantified in all samples with very good precision (Table 6). For one individual, 24,25(OH)_2_D_3_ levels were below LLOQ. The same person also exhibited the lowest 3β-25(OH)D_3_ concentrations. For most samples, levels of 1,25(OH)_2_D_3_ were below LLOQ.

**Table 6:** Measured concentrations of multiple vitamin D_3_ metabolites in plasma samples of healthy individuals

	3β-25(OH)D_3_		3α-25(OH)D_3_		24,25(OH)_2_D_3_		1,25(OH)_2_D_3_		Vitamin D_3_	
Sample	Concentration (ng/ml)	CV%	Concentration (ng/ml)	CV%	Concentration (ng/ml)	CV%	Concentration (ng/ml)	CV%	Concentration (ng/ml)	CV%
1	25.0	1.2	1.30	2.2	1.64	5.5	0.69	13.2	13.3	6.0
2	24.9	1.9	1.12	3.2	2.59	7.5	*<LLOQ*	—	7.4	5.7
3	54.4	2.0	2.99	2.0	5.92	2.1	0.57	13.0	12.5	2.4
4	7.4	2.8	0.29	3.0	*<LLOQ*	—	*<LLOQ*	—	4.3	6.6
5	35.8	3.7	1.90	3.0	3.90	1.7	*<LLOQ*	—	14.7	3.6
6	19.9	2.0	0.81	4.0	1.76	3.6	*<LLOQ*	—	4.9	7.6
7	25.1	2.3	0.94	3.5	1.77	3.4	*<LLOQ*	—	4.7	4.6
8	17.9	1.8	0.73	3.3	1.30	9.6	*<LLOQ*	—	3.8	11.1
9	22.1	2.7	1.16	3.8	2.36	5.0	*<LLOQ*	—	4.7	7.5
10	19.0	2.6	0.87	3.5	1.05	3.8	*<LLOQ*	—	7.8	7.5
11	14.1	3.2	0.49	3.6	1.36	7.7	*<LLOQ*	—	5.6	9.4
12	16.2	1.2	0.80	3.3	1.31	2.7	*<LLOQ*	—	4.9	8.2

## Discussion

It is shown in the Results section above that all products of the FMP-TS reaction were fully resolved for the investigated vitamin D metabolites under the chosen chromatographic conditions, including isomers and epimers.

We also demonstrated that not all -OH groups of the vitamin D compounds are amenable to the FMP-TS reaction. Complete reaction of all -OH groups using the pyridinium salt would result in precursor ions at [M+92]^+^, [M+184]^2+^, and [M+276]^3+^ for 1,25(OH)_2_D_3_ and 24,25(OH)_2_D_3_. However, [M+276]^3+^ was not obtained because one -OH group is a tertiary alcohol, which according to Quirke *et al.* (21) does not undergo the reaction. The difference between 1,25(OH)_2_D_3_ and 24,25(OH)_2_D_3_; viz., the formation of [M+184]^2+^ only for 24,25(OH)_2_D_3_, can be explained by stereochemical hindrance at C-1 and repulsive forces between FMP-cations in 1,25(OH)_2_D_3_ at C-1 and C-3 if both hydroxyl groups are attached. Similarly, 3α-25(OH)D_3_ and 3β-25(OH)D_3_ reacted only at one -OH group since the second -OH group is a tertiary alcohol. Vitamin D_3_ was derivatized at the single hydroxyl group, which is connected to a secondary carbon atom.

Most of the metabolites’ derivatization products exhibited the expected enhanced responses because of the permanent charge, which is generally beneficial for electrospray ionization response. The strongest gains were seen for the native vitamin D_3_, exhibiting significant 285-fold improvement of the peak areas. The smallest gains were observed for 1,25(OH)_2_D_3_ (3×). A possible explanation for the discrepancy between monohydroxylated and dihydroxylated metabolites (e.g., 24,25(OH)_2_D_3_ showed lower increases in relative peak areas than 25(OH)D_3_ epimers) is the formation of more than one product because of the two hydroxyl groups at C-3 and C-24. Similarly, Lin *et al.* reported that signal intensities of some of their investigated compounds were positively affected, whereas others were not. Specifically, signal intensities of 17β-estradiol-FMP, estrone-FMP, and 17α-ethinyl estradiol-FMP were 2.19–12.1 higher than the nonderivatized molecules, whereas estriol-FMP produced smaller signals than the nonderivatized metabolite (28). Again, the formation of multiple products after reaction of more than one of the three hydroxyl groups of the estriol molecule was the likely reason for the reduced signals. Furthermore, the authors observed that additives such as formic acid and the reconstitution procedure before measurement influenced ion suppression and thus final signal intensities.

We optimized the derivatization reaction further by studying the type of solvent and the presence of moisture in the reaction solvent; type of base and concentration, FMP-TS concentration, as well as incubation temperature and incubation time (see Results section). ACN was chosen for the reaction, based on literature data (28), as well as our further investigation with respect to the presence of water in the solvent. TEA was chosen over TMA as base to promote the reaction. Importantly, the base concentration exhibited a significant impact on the important 3α-25(OH)_2_D_3_ metabolite, which is commonly present at very low concentrations in serum. Beinhauer *et al.* (26) used FMP-TS at 25 mg/ml for determining trace levels of estrogens in serum. However, when we investigated FMP-TS concentrations higher than 5 mg/ml, negative effects were observed for 1,25(OH)_2_D_3_, 24,25(OH)_2_D_3,_ and vitamin D_3,_ and only 25(OH)D_3_ epimers were slightly positively affected (Fig. 2) by the increase. We chose to proceed with 5 mg/ml since 1,25(OH)_2_D_3_ is a metabolite of interest but present in very low abundance. This concentration allowed the measurement of both 3β-25(OH)D_3_ and 3α-25(OH)D_3_. Incubation temperature higher than 30^°^C had a negative impact on the majority of vitamin D_3_ metabolites. However, Faqehi *et al.* (27) observed higher reaction yields for estrogens using FMP-TS after increasing temperature from 25 to 40^°^C. Much higher incubation temperatures (80^°^C) were applied by Beinhauer *et al.* in the presence of sodium hydrogen carbonate (pH = 10) to derivatize estrone, α-estradiol and β-estradiol, and estriol in serum (26). The authors did not investigate incubation temperatures <60^°^C in their experiments.

Recovery of the method was very satisfactory for every metabolite. For the calibration curves, simple linear regression was chosen with or without weighted factor as the calibration model. Moreover, the method was accurate and precise both within-day as well as between-day measurements. In addition, extra care was taken to ensure quality of the measurement of 3β-25(OH)D_3_, thus commercial QC samples were used with satisfactory results. LODs and LLOQs demonstrated the suitability of the method for the application human serum samples, except for 1,25(OH)_2_D_3_, for which the LLOQ was too high, and thus, the method was not fit for purpose for 1,25(OH)_2_D_3_.

We also designed stability experiments for FMP-TS products, as there is only limited information in the literature. This is important, as in daily practice, there is always the possibility that the analysis of processed samples (including derivatization) cannot be completed within the planned time frame, for example, in the case of instrument failure. It may then be necessary to store these processed samples at low temperatures and run them the following day or even later. It is therefore important to determine the maximum length of time, for which processed samples can be preserved, before they undergo unacceptable degradation. This is particularly important for products of the chemical derivatization, as they may be less stable than the native compounds. Some stability data on FMP derivatization products have been previously reported for other analytes. Bald (25) investigated the stability of thiol-FMP products after a period of 2 weeks and reported no changes during electrophoretic separation, with no detailed information on potential quantitative losses. Fagehi *et al.* (27) found that FMP products of estrone and estradiol from plasma extracts were stable for over 24 h in the autosampler at 10^°^C for 24 h at −20^°^C (which agrees with our observations) and −80^°^C as well as for 28 days at −80^°^C. Significant degradation (−28% to −42%) of estrogen-FMP products was observed after 48 h at −20^°^C. Moreover, Beinhauer *et al.* (26) reported that estrone, α-estradiol and β-estradiol, and estriol FMP products in serum were stable for 8 h at room temperature and for 48 h at −20^°^C.

In our experiments, the FMP products in our study were stable at −20^°^C for short periods (24 h). For longer periods, lower temperature storage (−80^°^C) is required.

Finally, we successfully demonstrated the applicability of the developed method for human serum measurements of healthy individuals.

In conclusion, a simple, fast, and sensitive LC-MS/MS method was developed for comprehensive measurement of vitamin D_3_ metabolites after chemical derivatization with FMP-TS, which has not previously been used for vitamin D analysis. The derivatization reaction was initially optimized, and the assay was validated with excellent results. FMP-TS derivatization strongly enhanced the detection sensitivity of the investigated analytes because of the permanent positive charge, which was added to the molecule. At the same time, chromatographic separation of all investigated species was achieved, including full separation of the isomeric dihydroxylated species as well as the epimers of 25(OH)D_3_. Importantly, the described protocol is simpler, less expensive, and faster (approximately 15 min) than many derivatization protocols in the literature, which usually require shaking in a water bath, 1–3 h of incubation (22, 25, 28), or additional clean-up steps after the derivatization reaction (30).

Unfortunately, the developed method was not fit for the purpose for 1,25(OH)_2_D_3_ as levels of this metabolite in serum are usually lower than the LLOQ of our assay. To overcome this limitation for 1,25(OH)_2_D_3_, Amplifex reagent can be readily used (15, 31, 32); however, at much higher cost as compared with FMP-TS and longer assay run times.

## Data availability

The data that support the findings of this study are available from the corresponding author (Prof Dr Dietrich Volmer, dietrich.volmer@hu-berlin.de) upon reasonable request.

## Supplemental data

This article contains supplemental data.

## Conflict of interest

The authors declare that they have no conflicts of interest with the contents of this article.

## References

[1] Sempos C.T., Betz J.M., Camara J.E., Carter G.D., Cavalier E., Clarke M.W. (2017). General steps to standardize the laboratory measurement of serum total 25-hydroxyvitamin D. J. AOAC Int..

[2] Wise S.A., Phinney K.W., Tai S.S.C., Camara J.E., Myers G.L., Durazo-Arvizu R. (2017). Baseline assessment of 25-hydroxyVitamin D assay performance: a Vitamin D standardization program (VDSP) interlaboratory comparison study. J. AOAC Int..

[3] Durazo-Arvizu R.A., Tian L., Brooks S.P.J., Sarafin K.I., Cashman K.D., Kiely M. (2017). The Vitamin D standardization program (VDSP) manual for retrospective laboratory standardization of serum 25-hydroxyVitamin D data. J. AOAC Int..

[4] Zhang R., Naughton D.P. (2010). Vitamin D in health and disease: current perspectives. Nutr. J..

[5] Hagenhoff S., Hayen H. (2018). LC/MS analysis of vitamin D metabolites by dielectric barrier discharge ionization and a comparison with electrospray ionization and atmospheric pressure chemical ionization. Anal. Bioanal. Chem..

[6] Alexandridou A., Volmer D.A. (2022). Sample preparation techniques for extraction of vitamin D metabolites from non-conventional biological sample matrices prior to LC–MS/MS analysis. Anal. Bioanal. Chem..

[7] Alexandridou A., Schorr P., Volmer D.A. (2023). Comparing derivatization reagents for quantitative LC–MS/MS analysis of a variety of vitamin D metabolites. Anal. Bioanal. Chem..

[8] Ding S., Schoenmakers I., Jones K., Koulman A., Prentice A., Volmer D.A. (2010). Quantitative determination of vitamin D metabolites in plasma using UHPLC-MS/MS. Anal. Bioanal. Chem..

[9] Higashi T., Shibayama Y., Fuji M., Shimada K. (2008). Liquid chromatography – tandem mass spectrometric method for the determination of salivary 25-hydroxyvitamin D3: a noninvasive tool for the assessment of vitamin D status. Anal. Bioanal. Chem..

[10] Lipkie T.E., Janasch A., Cooper B.R., Hohman E.E., Weaver C.M., Ferruzzi M.G. (2013). Quantification of vitamin D and 25-hydroxyvitamin D in soft tissues by liquid chromatography-tandem mass spectrometry. J Chromatogr. B Analyt. Technol. Biomed. Life Sci..

[11] Zelzer S., Meinitzer A., Enko D., Simstich S., Le Goff C., Cavalier E. (2020). Simultaneous determination of 24,25- and 25,26-dihydroxyvitamin D3 in serum samples with liquid-chromatography mass spectrometry – a useful tool for the assessment of vitamin D metabolism. J Chromatogr. B Analyt. Technol. Biomed. Life Sci..

[12] Lyu H., Wang S., Jin Y., Shen R., Chen J., Zhu C. (2020). Simultaneous determination of VD2, VD3, 25(OH) D2, and 25(OH) D3 in human plasma using electrospray LC–MS/MS as well as its application to evaluate VD plasma levels in depressive, schizophrenic patients and healthy individuals. Biomed. Chromatogr..

[13] Bonnet L., Margier M., Svilar L., Couturier C., Reboul E., Martin J.C. (2019). Simple fast quantification of cholecalciferol, 25-hydroxyvitamin D and 1,25-dihydroxyvitamin D in Adipose Tissue using LC-HRMS/MS. Nutrients.

[14] Fabregat-Cabello N., Darimont P., Huyghebaert L., Reynier P., Annweiler C., Milea D. (2019). Liquid chromatography-tandem mass spectrometry for monitoring vitamin D hydroxymetabolites in human aqueous humor. Anal. Methods.

[15] Ivison F.M., Hinchliffe E., Howarth N., Pickersgill M., Tetlow L. (2019). Development of a mass spectrometry method for 1,25-dihydroxy vitamin D3 using immunoextraction sample preparation. Ann. Clin. Biochem..

[16] Ogawa S., Ooki S., Morohashi M., Yamagata K., Higashi T. (2013). A novel cookson-type reagent for enhancing sensitivity and specificity in assessment of infant vitamin D status using liquid chromatography/tandem mass spectrometry. Rapid Commun. Mass Spectrom..

[17] Higashi T., Homma S., Iwata H., Shimada K. (2002). Characterization of urinary metabolites of vitamin D3 in man under physiological conditions using liquid chromatography-tandem mass spectrometry. J. Pharm. Biomed. Anal.

[18] Abdel-Khalik J., Crick P.J., Carter G.D., Makin H.L., Wang Y., Griffiths W.J. (2014). Studies on the analysis of 25-hydroxyvitamin D3 by isotope-dilution liquid chromatography – tandem mass spectrometry using enzyme-assisted derivatisation. Biochem. Biophys. Res. Commun..

[19] Le J., Yuan T.-F., Geng J.-Q., Wang S.-T., Li Y., Zhang B.-H. (2019). Acylation derivatization based LC-MS analysis of 25-hydroxyvitamin D from finger-prick blood. J. Lipid Res..

[20] Higashi T., Suzuki M., Hanai J., Inagaki S., Min J.Z., Shimada K. (2011). A specific LC/ESI-MS/MS method for determination of 25-hydroxyvitamin D3 in neonatal dried blood spots containing a potential interfering metabolite, 3-epi-25-hydroxyvitamin D3. J. Sep. Sci..

[21] Quirke J.M.E., Adams C.L., Van Berkel G.J. (1994). Chemical derivatization for electrospray ionization mass spectrometry. 1. Alkyl halides, alcohols, phenols, thiols, and amines. Anal. Chem..

[22] Mukaiyama T., Ikeda S., Kobayashi S. (1975). A novel method for the preparation of various 2-pyridyl sulfides from alcohols. Chem. Lett..

[23] Scouten W.H., Rinesp R., Mulderp A.H.L., Reinhart L. (1988). Chromophoric and affinity ligand-containing halogenated N-alkyl pyridiniums as activating agents for hydroxylic matrices. Ann. N. Y Acad. Sci..

[24] McMurry J. (2002). Fundam. Org. Chem..

[25] Bald E. (1979). Analytical utility of 2-halopyridinium salts: i. Paper electrophoretic characterization of thiols as 2-alkyl (aryl) thio-1-methylpyridinium p-toluenesulphonates. J Chromatogr. B Analyt. Technol. Biomed. Life Sci..

[26] Beinhauer J., Bian L., Fan H., Šebela M., Kukula M., Barrera J.A. (2015). Bulk derivatization and cation exchange restricted access media-based trap-and-elute liquid chromatography – mass spectrometry method for determination of trace estrogens in serum. Anal. Chim. Acta.

[27] Faqehi A.M.M., Cobice D.F., Naredo G., Mak T.C.S., Upreti R., Gibb F.W. (2016). Derivatization of estrogens enhances specificity and sensitivity of analysis of human plasma and serum by liquid chromatography tandem mass spectrometry. Talanta.

[28] Lin Y.H., Chen C.Y., Wang G.S. (2007). Analysis of steroid estrogens in water using liquid chromatography/tandem mass spectrometry with chemical derivatizations. Rapid Commun. Mass Spectrom..

[29] Alexandridou A., Volmer D.A. (2023). Stability of sample extracts of vitamin - D3 metabolites after chemical derivatization for LC–MS/MS analysis. Anal. Bioanal. Chem..

[30] Higashi T., Yamauchi A., Shimada K. (2005). 2-Hydrazino-1-methylpyridine: a highly sensitive derivatization reagent for oxosteroids in liquid chromatography-electrospray ionization-mass spectrometry. J Chromatogr. B Analyt. Technol. Biomed. Life Sci..

[31] Müller M.J., Stokes C.S., Volmer D.A. (2016). Quantification of the 3α and 3β epimers of 25- hydroxyvitamin D3 in dried blood spots by LC-MS/MS using artificial whole blood calibration and chemical derivatization. Talanta.

[32] Hedman C.J., Wiebe D.A., Dey S., Plath J., Kemnitz J.W., Ziegler T.E. (2014). Development of a sensitive LC/MS/MS method for vitamin D metabolites: 1,25 Dihydroxyvitamin D2&3 measurement using a novel derivatization agent. J Chromatogr. B Analyt. Technol. Biomed. Life Sci..

