# Supplementary material for: 2-fluoro-1-methylpyridinium p-toluene sulfonate: a new LC-MS/MS derivatization reagent for vitamin D metabolites
Source: J Lipid Res. 2023 Jul 3;64(8):100409. doi: 10.1016/j.jlr.2023.100409 (PMC10410174; doi:10.1016/j.jlr.2023.100409)
Supplement: Supplemental Tables S1 and S2 and Figure S1 [file mmc1.docx]

*Supplementary material*

**2-fluoro-1-methylpyridinium p-toluene sulfonate: a new LC-MS/MS derivatization reagent for vitamin D metabolites**

**Anastasia Alexandridou^1^, Dietrich A. Volmer^1^***

**^1^***Bioanalytical Chemistry, Humboldt University Berlin, Brook-Taylor-Str. 2, 12489 Berlin, Germany*

**Authors’ ORCID**

*Anastasia Alexandridou: 0000-0003-3633-4021*

*Dietrich A. Volmer: 0000-0003-2820-1480*

**Funding sources**

DAV acknowledges research funding by the German Research Foundation (DFG VO 1355/5-2). AA is grateful for a supplementary research fellowship by the School of Analytical Sciences Adlershof (SALSA STF23-01).

*Corresponding author:

Prof. Dr. Dietrich Volmer

Humboldt University Berlin

Department of Chemistry

12489 Berlin, Germany

Tel +49 30 2093 7588

Email: Dietrich.Volmer@hu-berlin.de

**Table S1.** Optimized ion source and MRM settings for the vitamin D_3_ metabolite-FMP-products.

| **Ion source parameters** | | | | | | | |
| --- | --- | --- | --- | --- | --- | --- | --- |
| IonSpray voltage | | | 5500 V | | | | |
| Curtain gas | | | 40 psi | | | | |
| Source temperature | | | 350 ^o^C | | | | |
| Ion source gas 1 (nebulizer gas) | | | 45 psi | | | | |
| Ion source gas 2 (heating gas) | | | 40 psi | | | | |
| Collision gas pressure | | | Medium | | | | |
| **MRM settings** | | | | | | | |
| Compound | Q1 (*m/z*) | Q3 (*m/z*) | | DP (V) | EP (V) | CE (V) | CXP (V) |
| 3β-25(OH)D_3_-FMP / 3α-25(OH)D_3_-FMP | 492.3 | 383.2 | | 35 | 13 | 12 | 13 |
|  | 492.3 | 365.2 | | 35 | 13 | 18 | 13 |
|  | 492.3 | 159.1 | | 35 | 13 | 35 | 17 |
| 24,25(OH)_2_D_3_- FMP / 1,25(OH)_2_D_3_- FMP | 508.3 | 399.3 | | 35 | 13 | 13 | 15 |
|  | 508.3 | 381.3 | | 35 | 13 | 18 | 16 |
|  | 508.3 | 363.3 | | 35 | 13 | 20 | 13 |
| D_3_-FMP | 476.3 | 367.3 | | 30 | 13 | 14 | 12 |
|  | 476.3 | 159.1 | | 30 | 13 | 37 | 18 |
|  | 476.3 | 145.0 | | 30 | 13 | 40 | 17 |

*declustering potential (DP), entrance potential (EP), collision energy (CE) and collision cell exit potential (CXP)

**Table S2.** Short-term autosampler stability (24 h, 10 ^o^C) of FMP products of vitamin D_3_ metabolites in serum extract (the results are expressed relative to the values at *t* = 0).

| Analyte | Spiked concentration (ng/mL) | *t*=0 | Stability  24 h, 10 ^o^C |
| --- | --- | --- | --- |
| 24,25(OH)_2_D_3_ | 0.5 | 100 | 96 |
|  | 8 | 100 | 99 |
|  | 35 | 100 | 98 |
| 1,25(OH)_2_D_3_ | 0.5 | 100 | 109 |
|  | 5 | 100 | 104 |
|  | 9 | 100 | 106 |
| 3β-25(OH)D_3_ | 8 | 100 | 98 |
|  | 50 | 100 | 101 |
|  | 90 | 100 | 99 |
| 3α-25(OH)D_3_ | 0.5 | 100 | 95 |
|  | 8 | 100 | 95 |
|  | 18 | 100 | 95 |
| D_3_ | 8.7 | 100 | 93 |
|  | 50.7 | 100 | 93 |
|  | 90.7 | 100 | 94 |


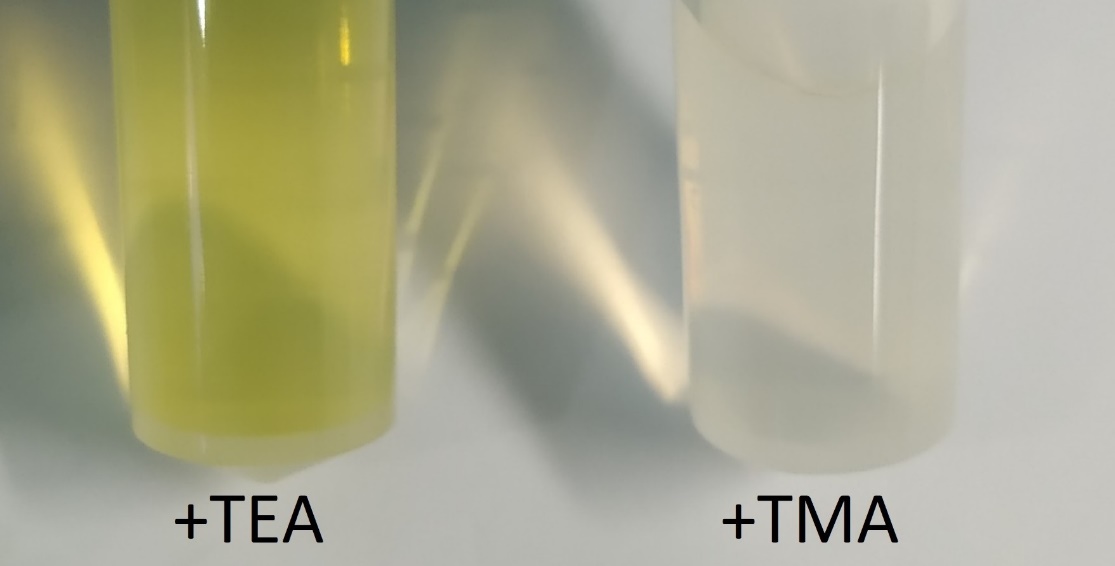


**Figure S1.** Optimization of the FMP-TS reaction after the addition of TEA (yellow) and TMA (clear) in dry acetonitrile (5 mg/mL), incubation at 40 ^o^C for 15 min.
